# Intestinal and systemic inflammation induced by symptomatic and asymptomatic enterotoxigenic *E. coli* infection and impact on intestinal colonization and ETEC specific immune responses in an experimental human challenge model

**DOI:** 10.1080/19490976.2021.1891852

**Published:** 2021-02-27

**Authors:** Jessica Brubaker, Xueyan Zhang, A. Louis Bourgeois, Clayton Harro, David A Sack, Subhra Chakraborty

**Affiliations:** a Department of International Health, Johns Hopkins Bloomberg School of Public Health, Johns Hopkins University, Baltimore, MD, USA; b PATH, Center for Vaccine Innovation and Access, Washington, DC, 20001, USA

**Keywords:** ETEC, intestinal inflammation, systemic inflammation, cytokines, immune response, myeloperoxidase, intestinal fatty acid-binding protein

## Abstract

Recent studies have gained a better appreciation of the potential impacts of enteric infections beyond symptomatic diarrhea. It is recognized that infections by several enteropathogens could be associated with growth deficits in children and intestinal and systemic inflammation may play an important underlying role. With enterotoxigenic *E. coli* (ETEC) being one of the leading causes of diarrhea among children in the developing world and important contributor to stunting, a better understanding of the impact of ETEC infection beyond diarrhea is timely and greatly needed. To address this, we evaluated if ETEC infection induces intestinal and systemic inflammation and its impact on colonization and immune responses to ETEC vaccine-specific antigens in a dose descending experimental human challenge model using ETEC strain H10407. This study demonstrates that the concentrations of myeloperoxidase (MPO) in stool and intestinal fatty acid-binding protein (an indicator of compromised intestinal epithelial integrity) in serum, significantly increased following ETEC infection in both diarrhea and asymptomatic cases and the magnitudes and kinetics of MPO are dose and clinical outcome dependent. Cytokines IL-17A and IFN-γ were significantly increased in serum post-ETEC challenge. In addition, higher pre-challenge concentrations of cytokines IL-10 and GM-CSF were associated with protection from ETEC diarrhea. Interestingly, higher MPO concentrations were associated with higher intestinal colonization of ETEC and lower seroconversions of colonization factor I antigen, but the reverse was noted for seroconversions to heat-labile toxin B-subunit. Together this study has important implications for understanding the acute and long-term negative health outcomes associated with ETEC infection.

## Introduction

Enterotoxigenic *Escherichia coli* (ETEC) is a leading bacterial cause of morbidity and mortality due to diarrhea in children in resource-poor settings.^1–3^ ETEC is also the most frequent cause of diarrhea in travelers and deployed military service members.^4–6^ Multiple recent studies have reaffirmed the importance of ETEC and indicated that afflicted children are more likely to have poor health outcomes.^7,8^

Although the death rate from diarrheal diseases has declined,^9^ the potential impacts of enteric infections beyond symptomatic diarrhea have become increasingly apparent.^10–12^ It is recognized that infections (diarrhea or asymptomatic), could lead to environmental enteric dysfunction (EED), malnutrition, deficits in growth and, could potentially impact cognitive development in the children of the developing world.^10,11,13,14^ Intestinal and systemic inflammation as well as intestinal gut barrier dysfunction due to enteric infections likely play an important underlying role in the mechanisms of EED and thereby contribute to the growth failure.^10,11,14^ Given the importance of ETEC, causing ~ 1 billion cases of diarrhea annually,^15^ better understanding of the consequences of ETEC infection and diarrhea is needed.

Studies have shown that children infected with ETEC are at higher risk of becoming stunted.^2,8,16–18,19^ However, the impact of ETEC infection on intestinal and systemic inflammation is not yet fully elucidated. Most of the previous studies were cross-sectional and thus it is difficult to determine the magnitude and kinetics of inflammation. The available data on the inflammation due to ETEC infection among children in the endemic countries are often confounded by co-infection(s) and their interactions, which also makes it difficult to ascertain the sole impact of ETEC on inflammation.^12,20,21^ In addition, if inflammation modifies intestinal colonization of ETEC, the impact of this inflammation on immune responses to ETEC needs to be studied.

In this study, we evaluated intestinal and systemic inflammation following ETEC infection in an experimental ETEC challenge model in humans. We determined if the magnitude and kinetics of inflammation depends on the infection dose, the clinical outcome, and the role of antibiotic treatment. We also studied if the degree of inflammation impacts the quantity of shedding of ETEC in stool and immune responses to ETEC vaccine-specific antigens. We used levels of myeloperoxidase (MPO) in stool as the indicator of intestinal inflammation and the serum levels of intestinal fatty acid-binding protein (I-FABP) as the indicator of compromised intestinal epithelial integrity. To determine systemic inflammation, a panel of serum pro-inflammatory cytokines were measured to study the post-challenge cytokine responses. We also evaluated any associations of pre-challenge cytokine levels in predicting the clinical outcome.

## Results

The details of the experimental challenge model study cohorts along with the stool and serum samples used in this study are described in the method section and the list of samples is given in Table 1.

**Table 1.** List of samples tested in this study

Type of samples/type of markers tested	No. of subjects evaluated					
	Cohort 1 Dose 10^8^ Subjects (n) = 9		Cohort 1 & 2 Dose 10^7^ Subjects (n) = 24		Cohort 4 Dose 10^6^/10^5^ Subjects (n) = 12 or (n) = 30	
	MSD	ND	MSD	ND	MSD	ND
**Stool/MPO**	9	0	18	6	6	6
**Serum/I-FABP**					6	6
**Serum/Cytokines**					6	24
**Post challenge shedding of ETEC data**			18	6		
**Serum and ALS/Immune responses data**			18	6		

### Magnitudes and kinetics of MPO following ETEC challenge

#### MPO concentrations depend on the clinical outcome following ETEC challenge

**Figure 1.**
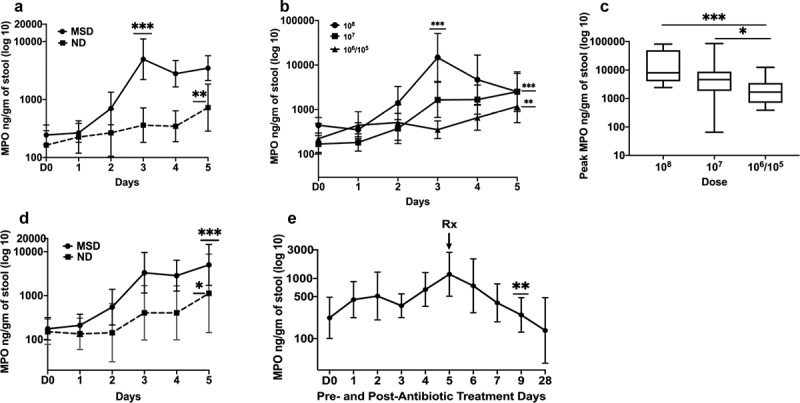
MPO concentrations pre- and post ETEC challenge

Among volunteers who had moderate to severe diarrhea (MSD) following experimental challenge with ETEC H10407 (all doses), the MPO concentrations in the stool increased significantly from the baseline (day before challenge) with the highest geometric mean (GM) fold increase of 20.2 fold (*p* < .0001) and peaked at GM 4958.6 ng/gm of stool on day 3 (Figure 1a). The concentrations of MPO on day 2 (2.87fold, *p* = .0100), day 4 (11.4fold, *p* < .0001) and day 5 (14.3fold, *p* < .0001) were also significantly higher than at baseline (Figure 1a).

A. Magnitudes and kinetics of MPO based on clinical outcome following ETEC challenge (all doses). B. Magnitudes and kinetics of MPO concentrations by dose. C. Comparison of the peak MPO concentrations on any day by dose. Doses: 10^8^ or 10^7^ or 10^6^/10^5^ CFU of ETEC. D. Comparison of magnitudes and kinetics of MPO concentrations by clinical outcome when challenged with the dose 10^7^ CFU of ETEC. MSD: moderate to severe diarrhea; ND: no diarrhea; D0: day before challenge; 1 to 5: 1 to 5 days after challenge. E. Reduction of MPO concentrations after antibiotic treatment. Rx: Antibiotic treatment; D0: day before challenge; 1 to 28: 1 to 28 days after challenge with 10^6^/10^7^ dose. *p* values are for comparisons between baseline and the peak in Fig A, B and D; In Fig E, *p* values are for comparisons between pre- and post-antibiotic days. *p* < .05:*; *p* < .01:**, *p* < .001:**

Among the subjects who had no diarrhea (ND) following ETEC challenge, the GM MPO concentrations significantly increased to 727.3 ng/gm of stool (4.45fold, *p* = .0029) but reached the peak late, on day 5 (Figure 1a). The MPO concentrations were 2.21 and 2.11 folds higher on day 3 and day 4, respectively, compared to the baseline. When the peak concentrations were compared, the GM MPO level among the MSD was 6.82 fold higher than ND (*p* = .0046) (Figure 1a).

#### MPO concentrations depend on the challenge doses of ETEC

When the MPO concentrations were compared among the subjects infected with 10^8^, 10^7^ or 10^6^/10^5^ CFU doses of ETEC, a dose-dependent pattern was observed (Figure 1b). Among the subjects infected with 10^8^ dose, the GM of MPO reached the peak at 14,759.3ng/gm of stool (33.2 fold increase from baseline, *p* = .0002) on the day 3 post challenge while the GM of MPO reached the peak late on day 5 among the subjects infected with 10^7^ (2518.01ng/gm, fold increase 15, *p* < .0001) and 10^6^/10^5^ (1177.5ng/gm, fold increase 5.31, *p* = .0012). The GM MPO level was significantly higher on day 3 of 10^8^ dose compared to day 5 of 10^7^ dose (fold difference 5.86, *p* = .0063) which was 2.14 fold higher compared to day 5 of 10^6^/10^5^ dose but the difference was not significant (Figure 1b). When the highest concentrations of MPO on any day following ETEC challenge were evaluated the GM MPO of 10^8^ dose was 2.96 fold higher than 10^7^ dose but was not significantly different while it was 7.44 fold higher than 10^6^/10^5^ dose (*p* = .0007) (Figure 1c). GM MPO of 10^7^ dose was significantly higher than 10^6^/10^5^ dose (2.51fold *p* = .0295).

#### MPO concentrations vary by the clinical outcome within the same dose

We compared the MPO concentrations among subjects challenged with 10^7^ dose of ETEC that developed MSD with those in subjects who did not develop diarrhea. (Figure 1d). The GM MPO level in the subjects with MSD increased 28.6 fold and reached the peak (5016ng/gm of stool, *p* < .0001) on day 5 while it increased 7.49 fold (1126.9 ng/gm of stool, *p* = .0513) among the subjects with ND. Though the GM MPO level was higher for those with MSD on day 5, the difference when compared to those with ND was not significant. (*p* = .1375) (Figure 1d).

In the experimental challenge model, all the subjects were treated with antibiotics on day 5 (120 hours) after challenge if early antibiotic treatment was not required. In the low dose (10^6^/10^5^) cohort, the stool samples were collected and stored on extended days 6, 7, 9 and 28 days after challenge. Following the antibiotic treatment there was a reduction noted in the concentrations of MPO, 1.56 fold on day 6, 4.11 fold on day 7 and was significantly reduced by day 9 (4.75 fold, *p* = .0042) (Figure 1e). The stool samples after day 5 were not available from the higher dose cohorts.

### MPO level is associated with the shedding of ETEC in stool

The CFU of ETEC shed in the stool from the day after challenge everyday till the subjects were cleared of ETEC were estimated and correlated with the concentrations of MPO. The maximum concentrations of MPO on any day following challenge was positively correlated with the maximum CFU of ETEC in stool any day (ρ = 0.747, *p* < .008).

### Pre-challenge MPO concentrations are associated with immune responses to ETEC vaccine-specific antigens

**Figure 2.**
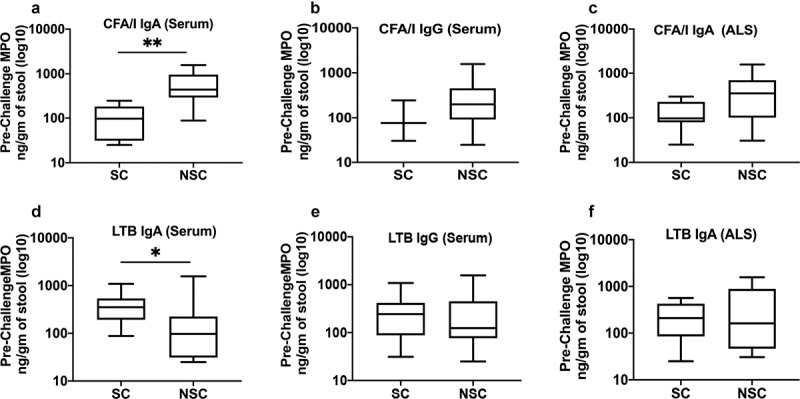
Pre-challenge MPO concentration in the subjects who did versus who did not seroconverted following challenge. Upper panel CFA/I, lower panel LTB; A,D: serum IgA, B,E: Serum IgG; C,F: ALS IgA. SC: seroconverted, NSC: not seroconverted; Box plot showing Min, Max and Median. *p* < .05:*; *p* < .01:**

The post challenge antibody titers to IgA and IgG of colonization factor antigen I (CFA/I) and heat labile toxin B subunit (LTB) in serum and IgA in antibody in lymphocyte supernatant (ALS) correlated with the concentrations of MPO on the day before challenge. Concentrations of pre-challenge MPO by seroconversion status are shown in Table 2 and Figure 2a-f. For CFA/I antigen, the subjects who did not seroconvert had higher concentrations of pre-challenge MPO than the subjects who did seroconvert both in serum (IgA 5.2 fold higher, *p* = .0014; IgG 2.5 fold higher) and in ALS (IgA 2.4 fold higher, *p* = .0831), (Figure a-c, Table 2). Interestingly, for LTB IgA in serum, the opposite trend was seen; the subjects who seroconvert had higher median concentrations of pre-challenge MPO (3 fold higher, *p* = .0434) compared to the subjects who did not seroconvert (Figure d, Table 2). The pre-challenge MPO levels were similar for LTB IgG in serum and LTB IgA in ALS, among the seroconverted and not seroconverted groups (Figure e-f, Table 2). When the immune responses to ETEC antigens was treated as a continuous variable (highest fold change of titers on any day), CFA/I titers negatively correlated with pre-challenge MPO concentrations but positively correlated with seroconversions to LTB with the exception of LTB IgG in serum which was negatively correlated (Table 3).

**Table 2.** Pre-challenge MPO concentrations in the subjects who did versus who did not seroconvert following challenge

**Antigen**	**Not seroconverted** GM (Range)	**Seroconverted** GM (Range)	**p value**
**Serum IgA**			
CFA/I	438.9(88.1–1566.6)	84.4(25–243.9)	0.0014
LTB	107.2(25–1566.6)	325.4(88.1–1086.4)	0.0434
**Serum IgG**			
CFA/I	204(25–1566.6)	83(30.6–243.9)	0.3422
LTB	158.9(25–1566.6)	205.4(31.3–1086.4)	0.9544
**ALS IgA**			
CFA/I	256.6(30.6–1566.6)	109.3(25–299.5)	0.0831
LTB	179.2(30.6–1566.6)	172.7(25–566.2)	1

GM: Geometric mean

**Table 3.** Correlations between pre-challenge MPO concentrations and highest fold changes of antibody titers following challenge

Sample type	Antigen	ρ (p value)*
**Serum IgA**	CFA/I	−0.781 (0.005)
	LTB	0.608 (0.047)
**Serum IgG**	CFA/I	−0.497 (0.042)
	LTB	−0.81 (0.008)
**ALS IgA**	CFA/I	−0.527 (0.03)
	LTB	0.605 (0.049)

*Spearman’s correlation coefficient *ρ* (*p* value)

### Post-challenge MPO concentrations are associated with immune responses to ETEC vaccine-specific antigens

Similar to the pre-challenge MPO, the post challenge highest MPO concentrations on any day was negatively correlated with the highest fold changes of CFA/I antigen in serum and ALS but positively correlated with LTB titers. (Table 4, *Supplement* Table S1).

**Table 4.** Correlations between post-challenge highest MPO concentrations and highest fold changes of antibody titers following challenge

Sample type	Antigen	ρ (p value)*
**Serum IgA**	CFA/I	−0.589 (0.046)
	LTB	0.489 (0.046)
**Serum IgG**	CFA/I	−0.526 (0.03)
	LTB	0.543 (0.024)
**ALS IgA**	CFA/I	−0.61 (0.046)
	LTB	0.605 (0.049)

*Spearman’s correlation coefficient *ρ* (*p* value)

### Serum I-FABP concentrations increased following ETEC challenge

**Figure 3.**
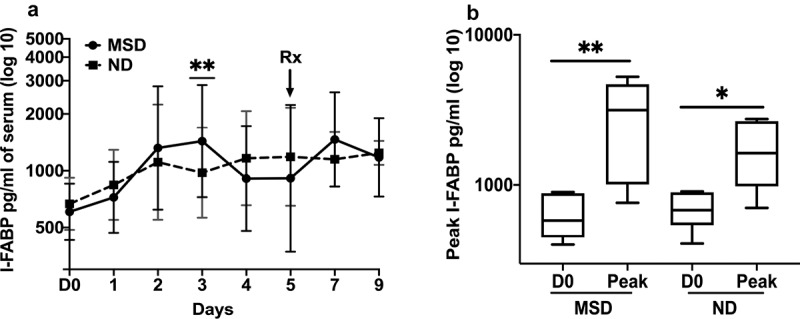
I-FABP concentrations pre- and post ETEC challenge

Following challenge with 10^6^/10^5^ doses of ETEC, although the day of the peak concentrations varied between the subjects (ranging from day 2 to day 4) and therefore the 95% confidence intervals were large, however, the GM of I-FABP concentrations significantly peaked on day 3 among the MSD subjects (*p* = .0087) while it peaked on day 5 (*p* = .1320) among the ND subjects (Figure 3a). The GM of the peak concentrations of I-FABP was 3.87 fold (*p* = .0087) and 2.33 fold (*p* = .0152) higher from the baseline among the MSDs and NDs, respectively (Figure 3b). The peak concentrations of I-FABP among the MSDs and NDs were not significantly different (fold difference 1.67, *p* = .1784). Antibiotic treatment on day 5 did not decrease the I-FABP concentrations (Figure 3a).

A. Magnitudes and kinetics of MPO based on clinical outcome following ETEC challenge with 10^6^/10^7^ dose. MSD: moderate to severe diarrhea; ND: no diarrhea; D0: day before challenge; 1 to 9: 1 to 9 days after challenge. B. Comparison of the pre- and post-challenged peak I-FABP concentrations on any day by clinical outcome. D0: day before challenge; Peak: highest concentrations of MPO any day; MSD: moderate to severe diarrhea; ND: no diarrhea; Box plot showing Min, Max and Median. *p* < .05:*; *p* < .01:**. Rx: Antibiotic treatment.

### Serum pro-inflammatory cytokines interleukin (IL)-17A and interferon (IFN)-γ increased following ETEC challenge

**Figure 4.**
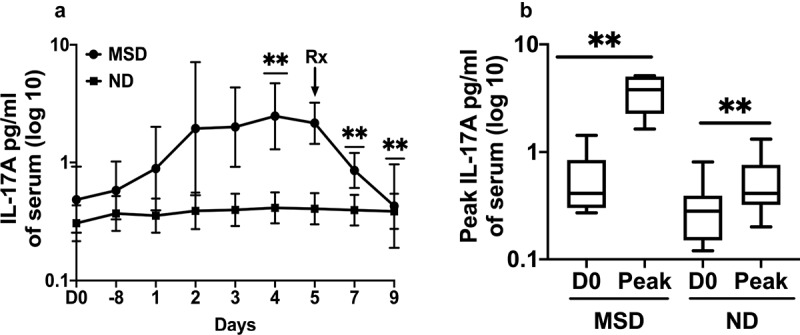
IL-17A concentrations pre- and post ETEC challenge. A. Magnitudes and kinetics of IL-17A concentrations by clinical outcome. MSD: moderate to severe diarrhea; ND: no diarrhea; D0: day before challenge; −8: 8 hours after challenge; 1 to 9: 1 to 9 days after challenge. B. Comparisons of the peak IL-17A concentrations on any day among the subjects with MSD and ND. D0: day before challenge; Peak: highest concentrations of IL-17A. Box plot showing Min, Max and Median. *p* < .01:**. Rx: Antibiotic treatment

Among the 11 pro-inflammatory cytokines tested, IL-17A significantly increased from the baseline following ETEC challenge with 10^6^/10^5^ dose (Figure 4a). Among the subjects with MSD, the GM of IL-17A increased 5.11 fold (*p* = .0012) and reached the peak on day 4. However, IL-17A concentrations remained close to baseline over the period among the subjects with ND (Figure 4a). The peak concentrations of IL-17A on any day among the MSDs was 7-fold higher (*p* = .0032) from the baseline (Figure 4b) and among the NDs was 1.73fold higher (*p* = .0043) from the baseline.

**Figure 5.**
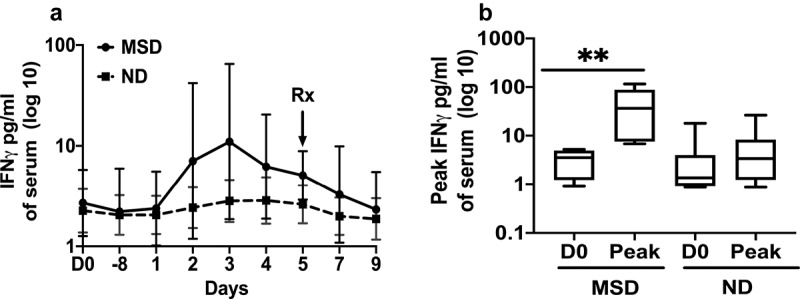
IFN-γ concentrations pre and post ETEC challenge.A. Magnitudes and kinetics of IFN-γ concentrations by clinical outcome. MSD: moderate to severe diarrhea; ND: no diarrhea; D0: day before challenge; D1-8: 8 hours after challenge; D1 to D9: 1 to 9 days after challenge. B. Comparisons of the peak IFN-γ concentrations on any day among the subjects with MSD and ND. D0: day before challenge; Peak: highest concentrations of IFN-γ. Box plot showing Min, Max and Median. *p* < .01:**. Rx: Antibiotic treatment

Although the day when the concentrations peaked, varied among the subjects, and therefore the 95% confidence intervals were larger, the GM of IFN-γ increased 4.08 fold (*p* = .0931) from the baseline and reached the peak on day 3 among the MSDs, while among the NDs it did not increase more than 1.27 fold in the follow up period (Figure 5a). The peak GM concentrations of IFN-γ on any day was 10.6 fold higher (*p* = .0022) than the baseline among the MSDs and 1.51fold higher (*p* = .0563) than the baseline among the NDs (Figure 5b). The levels of the other cytokines tested, granulocyte-macrophage colony-stimulating factor (GM-CSF), IL-1β, IL-2, IL-6, IL-4, IL-8, IL-10, IL-12p70, and tumor necrosis factor (TNF)-α didn’t increase significantly from the baseline even among the volunteers who had MSD. The kinetics of these cytokines among the subjects with MSD are shown in the Supplement Figure S1.

Following antibiotic treatment on day 5 among the subjects challenged with 10^6^/10^5^ dose, the IL-17 level decreased significantly by day 7 (2.52 fold, *p* = .0022) and further decreased on day 9 (5 fold, *p* = .0013) (Figure 4a). The IFN-γ level decreased 2.2 fold on day 9 following antibiotic treatment (Figure 5a).

### Pre-challenge concentrations of IL-10 and GM-CSF are associated with the post-challenge clinical outcome of the ETEC infection

**Figure 6.**
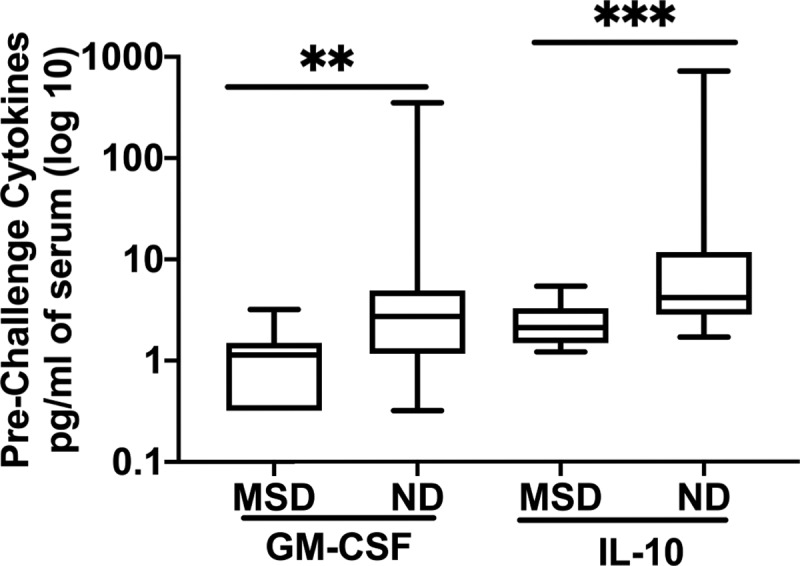
Pre-challenge concentrations of GM-CSF and IL-10 among the subjects with MSD or ND. MSD: moderate to severe diarrhea; ND: no diarrhea; Box plot showing Min, Max and Median. *p* = <0.01: **; *p* = <0.001: ***

We studied the concentrations of MPO, I-FABP and cytokines from the day before challenge among the subjects who had post-challenge MSD or ND. The pre-challenge GM concentrations of cytokine IL-10 and GM-CSF among the NDs were 3.35 fold (*p* = .0008) and 3.56 fold (*p* = .0064) higher, respectively, than among the MSDs (Figure 6). There were no significant associations of the pre-challenge concentrations of other inflammatory markers with the clinical outcome (Supplement Figure S2).

## Discussion

This study reports ETEC induced inflammation in an experimental human challenge model. Our study showed that ETEC infection significantly induces intestinal and systemic inflammation both among subjects with MSD and even among those who remained asymptomatic. This study is unique, since we could measure inflammatory markers among subjects who were likely not co-infected with any other enteric pathogens during the study and were challenged with a single strain of ETEC with a known dose in an inpatient unit. This controlled environment provided us a unique opportunity to determine the dose-dependent magnitude and kinetics of the inflammatory markers induced solely by ETEC in the longitudinally collected samples.

MPO has previously shown linear relation to the number of neutrophil cells and has been demonstrated to be elevated in the populations with enteropathy and associated with the acquisition of linear growth defects in the children from the endemic countries.^20,22–24^ MPO was also increased significantly following ETEC infection in a recent murine model study.^25^ In our study, following ETEC challenge, MPO in stool increased significantly, representing highly increased neutrophil activity at the level of the intestine. The magnitude of change in MPO concentration was dependent on clinical outcome and dose.

In this study, serum I-FABP, a marker of enterocyte death and gut barrier damage was significantly increased following even low dose of ETEC infection. Increased levels of I-FABP was previously shown to be associated with stunted children in Bangladesh.^26^ All these results signify the critical negative impact of ETEC infection and diarrhea on gut health.

An important insight of our study is the extent to which asymptomatic ETEC infection influences gut pathology. Interestingly, similar to the subjects who had MSD, the level of MPO and I-FABP were significantly increased following ETEC infection among the subjects with ND and the concentrations although lower than MSDs but were not significantly different at their highest peaks. This suggests that even asymptomatic ETEC infections could cause significant intestinal inflammation which adds further pathogen-specific evidence to the increasingly recognized effects of asymptomatic infections on the health of children in the developing world.

Our study highlighted the correlation of higher MPO concentrations with higher magnitude of post challenge fecal shedding of ETEC which represents increased colonization and replication of ETEC in the intestine. Notably, evidence shows that pathogens belonging to the family of Enterobacteriaceae utilize virulence factors to promote intestinal inflammation, which in turn confers a growth advantage for the pathogens in the gut lumen.^27,28^

The higher MPO concentrations in the gut also impact the immune responses of the host, as in our study, the seroconversion to CFA/I IgA was significantly lower in the subjects with higher pre-challenge as well as post-challenge MPO concentrations. It should be noted that colonization factors are the primary protective antigens in the current ETEC vaccine candidates. The children in the endemic countries, generally have higher levels of intestinal inflammations^29^ which could dampen the protective immune responses to the oral ETEC vaccines given. Similar results of association of MPO and EED with underperformance of rotavirus and polio vaccines were shown in Bangladeshi and Nicaraguan infants.^30,31^ Interestingly, in contrast, responses to LTB were higher with higher concentrations of MPO. LTB is immunomodulatory and may act differently, or alternatively, the lack of immunity to CFA/I may allow for greater exposure to LT.

Post ETEC challenge, IL-17A and IFN-γ were significantly increased in this study, which likely participates in inducing immune responses and clearance of ETEC from the gut. Th17 cells and their production of IL-17A have been implicated in the protection of the host against extracellular pathogens through induction of sIgA.^32,33^ IL-17A also has a potential role in clearing ETEC infection in piglets^34^ which could be associated with the heat-labile toxin, as the mucosal adjuvant double mutant heat-labile toxin, LT(R192G/L211A) or dmLT was shown to have the ability to enhance human Th17 responses.^35,36^

A well-recognized feature of infectious diseases is that their clinical course and eventual outcome can vary substantially between infected individuals. In a controlled experimental challenge study, this variability in disease prognosis would largely depend upon the host factors. A better understanding of the determinates of disease outcome could provide unique insights into disease biology as well as potentially revealing new therapeutic targets. Our study showed that higher pre-challenge concentrations of IL-10 and GM-CSF cytokines could be protective in ETEC diarrhea. IL-10 is an anti-inflammatory cytokine produced by dendritic cells, macrophages, and T-helper lymphocytes that downregulates the production of inflammatory mediators.^37^ It has been shown that IL-10^−/−^ mice are susceptible to continued inflammation as a result of interactions with endogenous gut bacteria at the bowel wall and that in the absence of IL-10 there is an unchecked pro-inflammatory response to luminal organisms.^38^ GM-CSF is a white blood cell growth factor which stimulates stem cells to produce granulocytes and macrophages, a process crucial for development of the immune system to promote defense against infections.^39^ Further studies are needed to understand any specific interactions of these cytokines with ETEC and ETEC toxins.

Intestinal and systemic inflammation leading to EED may be a valuable interventional target for programs aiming to reduce childhood morbidity in low and middle-income countries. As yet, there are no proven interventions against EED. Antibiotics act directly by preventing infections and may act indirectly by reducing subclinical infections and inflammation. In this study, all the subjects were treated with antibiotics on day 5, and this was followed by a significant reduction in the level of MPO. This could be showing the potential benefits of antibiotic treatment in reducing intestinal inflammation. The pro-inflammatory cytokines IL-17A and IFN-γ were also decreased following antibiotic treatment. It should be noted that since all the subjects in this study were treated with antibiotics on day 5 or earlier (if required) there was no control group who did not receive antibiotics; thus, it is difficult to conclude that the observed decrease in MPO and cytokines is a consequence of antibiotic treatment or the natural course of disease progression.

This study has limitations. Due to the unavailability of longitudinal serum samples from higher dose cohorts we were not able to test I-FABP and cytokines in serum in these cohorts and therefore unable to comment on the magnitude and kinetics following higher doses as well as dose-dependent variations of these inflammatory markers. A limitation of the MPO test is that the direct measured fecal concentrations of proteins in stools may vary depending on the relative concentration of water in the diarrhea (watery) or normal stool. However, it has been shown that there is significant co-relation between the stool weight normalized and protein normalized MPO concentrations in samples, suggesting a minimal need for protein normalization of these fecal biomarkers.^22^

In conclusion, our findings demonstrated the significant negative impact of ETEC diarrhea as well as asymptomatic infection on intestinal inflammation and gut barrier function and the sequel of events associated with the inflamed gut – higher colonization and replication of ETEC as well as decreased immune responses to ETEC colonization factor. In addition, our study elaborated the potential role of cytokines in protection against ETEC diarrhea and possible involvement in post-infection immune responses. These results have important implications for understanding the full negative potentials of ETEC infection. Our study reinforces the need for ETEC vaccines or other therapeutics to reduce ETEC disease burden and improve survival and long-term developmental potential in the children.

## Materials and methods

### Regulatory approval

The clinical study protocols,^40,41^ the experimental challenge model of ETEC was conducted under BB-IND 12,243 at the Center for Immunization Research (CIR), Johns Hopkins Bloomberg School of Public Health (JHBSPH). Approval to conduct the study was provided by the Western Institutional Review Board (Olympia, WA) for JHBSPH and PATH and by the Institutional Biosafety Committee of the Johns Hopkins Institutions. The study was registered in clinicalTrials.gov under NCT00844493.

### Experimental challenge model

In a dose descending experimental challenge model, healthy American adult volunteers were challenged with a dose of 10^8^, 10^7^, 10^6^ and 10^5^ CFU of an ETEC strain H10407 in 3 cohorts in an in-patient unit at Johns Hopkins University as described before.^40,41^ The challenge strain H10407 ETEC serotype O78:H11, produces LT and two forms of heat-stable toxin (STh and STp) and CFA/I. There were no placebo recipients in the study. Subjects were excluded if they had significant medical problems; if an HIV-1, hepatitis B, or hepatitis C test was positive; or if they had traveled to countries where ETEC or cholera infection is endemic within 2 years prior to receipt of investigational agent. After challenge, subjects were monitored for signs and symptoms of enteric illness. All the subjects were treated with antibiotics 120 hours (5 days) after challenge, or earlier if required because of the diarrhea illness according to the clinical protocol. Diarrhea was classified as mild (1 to 3 diarrheal stools totaling 200 to 400 g/24 h), moderate (4 to 5 diarrheal stools or 401 to 800 g/24 h), or severe (6 or more diarrheal stools or ≥800 g/24 h). No diarrhea was defined as no loose stool observed. Diarrheal attack rates (MSD) were similar across cohorts when same dose was used.^40^ The volunteers challenged with 2 × 10^8^ dose (cohort 1) resulted in attack rate (MSD) of 100%, and the volunteers challenged with 2 × 10^7^ dose (cohort 1 and 2) resulted in attack rate of 67 to 80%.^40^ The attack rate was 13.3% and 26.7% in the 10^5^ and 10^6^ groups, respectively, in cohort 4.^41^

### Sample selection for the current study

The available stool and serum samples collected and stored at −80°C during the challenge studies were used in the current study to evaluate MPO, I-FABP and cytokines. The details of the serum and stool samples and the associated challenge doses are described in Table 1. Stool samples from the day before challenge and day 1 to 5 after challenge in 10^8^ and 10^7^ doses and in addition day 6, 7 and 9 were used in 10^6^ and 10^5^ doses for testing MPO. Since there were only 4 subjects in 10^6^ dose and 2 subjects in 10^5^ dose who had (MSD), these two doses were combined for analysis in the current study. Since there were very few volunteers who had mild diarrhea, the samples from these volunteers were not included in this study. Longitudinal serum samples from the volunteers were only available from the 10^6^/10^5^ cohort and were used for I-FABP and cytokine analysis. Since there were higher number of subjects challenged with 2x10^7^dose and the attack rate (rates of MSD) was also higher, the analysis of the association of shedding and immune responses with inflammatory markers were done only in that dose. In addition, based on the prior studies, it was concluded that a dose of 2 × 10^7^ H10407 is the lowest practical dose for use in volunteer studies evaluating candidate vaccines and other preventive or therapeutic ETEC interventions.

The shedding and immune responses to ETEC antigens data were obtained from the previous published challenge studies.^40–42^ In short, qualitative and quantitative microbiology assessment were done to detect post-challenge shedding levels of the challenge strain ETEC H10407 in fecal samples. For qualitative assessment, up to 5 colonies appearing to be *E. coli* on MacConkey agar plates were tested for agglutination with antiserum to H10407. For semiquantitative microbiology, the fecal sample was serially diluted and spread onto MacConkey agar. After overnight incubation, the concentration of bacteria which appeared to be *E. coli* was calculated, and the proportion of these colonies (of 5 colonies tested) which agglutinated with anti-H10407 antisera was recorded.

ELISAs with serum and ALS samples were performed according to standard protocols.^40–43^ Titers were calculated to interpolate the dilution of serum, which yielded an optical density (OD) above baseline of 0.2 for serum samples and of 0.4 for ALS samples.^40,41^

### Evaluation of inflammatory markers:

MPO concentrations were measured in the stool samples by ELISA (Immunediagnostik, KR6603) following the manufacturer’s protocol. Samples were diluted 1:500 in kit-supplied wash buffer before being added to the plate. Results were calculated using a standard curve prepared using kit-supplied standards. MPO data were adjusted to the weight of the stool.

I-FABP concentrations were measured in serum by ELISA (R&D Systems, Inc., DFBP20) following the manufacturer’s protocol. Serum samples were diluted in a kit supplied diluent and added to the plates. Results were calculated using a standard curve prepared using kit-supplied standards.

For measuring cytokine concentrations in serum, the ultrasensitive human pro-inflammatory 10-plex and the ultrasensitive human IL −17A single-plex assays from Meso-Scale Discovery (MSD), Gaithersburg, MD were used. Blood serum was tested for eleven pro-inflammatory cytokines, GM-CSF, IL-1β

IL-2, IL-6, IL-4, IL-8, IL-10, IL-12p70, IL-17A, TNFα and IFN-γ, day before challenge and 8, 24, 48 and 72 hours and on 4, 5, 6, 7 and 9 days after challenge. The MSD multispot array was run according to the manufacturer’s protocol, with minor modifications.^43^ All samples were run in duplicate. Briefly, plates were preincubated with 25 μl of supplied human serum diluents. Calibration curves were prepared in the diluents as per manufacturer’s protocol. Following the 30-min incubation period, 25 μl of serum sample or calibrator was added to the wells. Plates were then washed and incubated with 25 μl of detection antibody for 2 h. Plates were read using the MS2400 imager (MSD). The lowest limit of quantification (LLOQ) was defined as the lowest calibrator value at which the coefficient of variance of concentration was less than 25% and recovery of calibrator was within 25% of the expected value. All cytokine values that were below the LLOQ were considered undetectable and assigned a value equal to the plate-specific LLOQ for statistical analyses.

### Statistical analysis

The day before challenge was considered as the baseline for all the analysis. Fold changes of the inflammatory markers were calculated as the ratio of post challenge days divided by the baseline. To determine differences between groups, Fishers Exact Test or chi-square was used as appropriate for categorical data, and for continuous outcomes t-test was used if the data in each group were normally distributed; if non-normally distributed, then Mann-Whitney-U test was employed. Seroconversion was defined as >2 fold and >4 fold increase from baseline of IgA and IgG titers to CFA/I and LTB antigens in serum and ALS, respectively. Fisher exact tests or Wilcoxon rank sum tests were used as appropriate to compare concentrations of MPO between subjects who did versus did not seroconvert following challenge. Spearman’s rank correlation coefficient (ρ) was estimated to examine associations between concentrations of MPO and fold changes in CFA/I and LTB IgA and IgG titers. All inflammatory markers were log‐transformed before analysis. A probability of <0.05 was considered statistically significant and the strength of association was determined by the coefficient values and their 95% confidence intervals (CIs). All analyses were performed using STATA version 13.0 IC (College Station, Texas) or Graph Pad Prism (GraphPad, CA) software.

## Supplementary Material

Supplemental Material
