# Supplementary material for: Intestinal and systemic inflammation induced by symptomatic and asymptomatic enterotoxigenic E. coli infection and impact on intestinal colonization and ETEC specific immune responses in an experimental human challenge model
Source: Gut Microbes. 2021 Feb 27;13(1):1891852. doi: 10.1080/19490976.2021.1891852 (PMC7919917; doi:10.1080/19490976.2021.1891852)
Supplement: Supplemental Material [file KGMI_A_1891852_SM3986.zip › Supplementary information/Revised Supplement Data.docx]

**Supplement data**

Table 1. Post-challenge highest MPO concentrations in the subjects who did versus who did not seroconverted following challenge

| **Antigen** | **Not seroconverted**  GM (Range) | **Seroconverted**  GM (Range) | **p value** |
| --- | --- | --- | --- |
| **Serum IgA** | | | |
| CFA/I | 17295 (1837.9-75600) | 3382.3 (124.5-85243.6) | 0.1201 |
| LTB | 3674.4 (124.5-69300) | 15593.5 (1837.9-85243.6) | 0.0882 |
| **Serum IgG** |  |  |  |
| CFA/I | 8324.7 (124.5-85243.6) | 2906.1(1853.7-4010.3) | 0.1642 |
| LTB | 5606.4 (124.5-69300) | 9868.9 (1837.9-85243.6) | 0.5811 |
| **ALS IgA** | | | |
| CFA/I | 6264.7 (124.5-75600) | 8004 (1853.7-85243.6) | 0.8958 |
| LTB | 2499.1 (124.5-11296.1) | 15897.1 (1853.7-85243.6) | 0.043 |

GM: Geometric mean
