# Supplementary material for: Intestinal and systemic inflammation induced by symptomatic and asymptomatic enterotoxigenic E. coli infection and impact on intestinal colonization and ETEC specific immune responses in an experimental human challenge model
Source: Gut Microbes. 2021 Feb 27;13(1):1891852. doi: 10.1080/19490976.2021.1891852 (PMC7919917; doi:10.1080/19490976.2021.1891852)
Supplement: Supplemental Material [file KGMI_A_1891852_SM3986.zip › Supplementary information/Supplement Figure content.docx]

**Supplement Figures**

**Supplement Figure 1.** Pro-inflammatory cytokine concentrations pre- and post ETEC challenge among the subjects with MSD. D0: day before challenge; -8: 8 hours after challenge; 1 to 9: 1 to 9 days after challenge.

**Supplement Figure 2.** Comparisons of pre-challenge concentrations of pro-inflammatory cytokines among the subjects with MSD (black) and ND (gray). MSD: moderate to severe diarrhea; ND: no diarrhea. Box plot showing Min, Max and Median.
