# Supplementary figures and images for: Intestinal and systemic inflammation induced by symptomatic and asymptomatic enterotoxigenic E. coli infection and impact on intestinal colonization and ETEC specific immune responses in an experimental human challenge model
Source: Gut Microbes. 2021 Feb 27;13(1):1891852. doi: 10.1080/19490976.2021.1891852 (PMC7919917; doi:10.1080/19490976.2021.1891852)

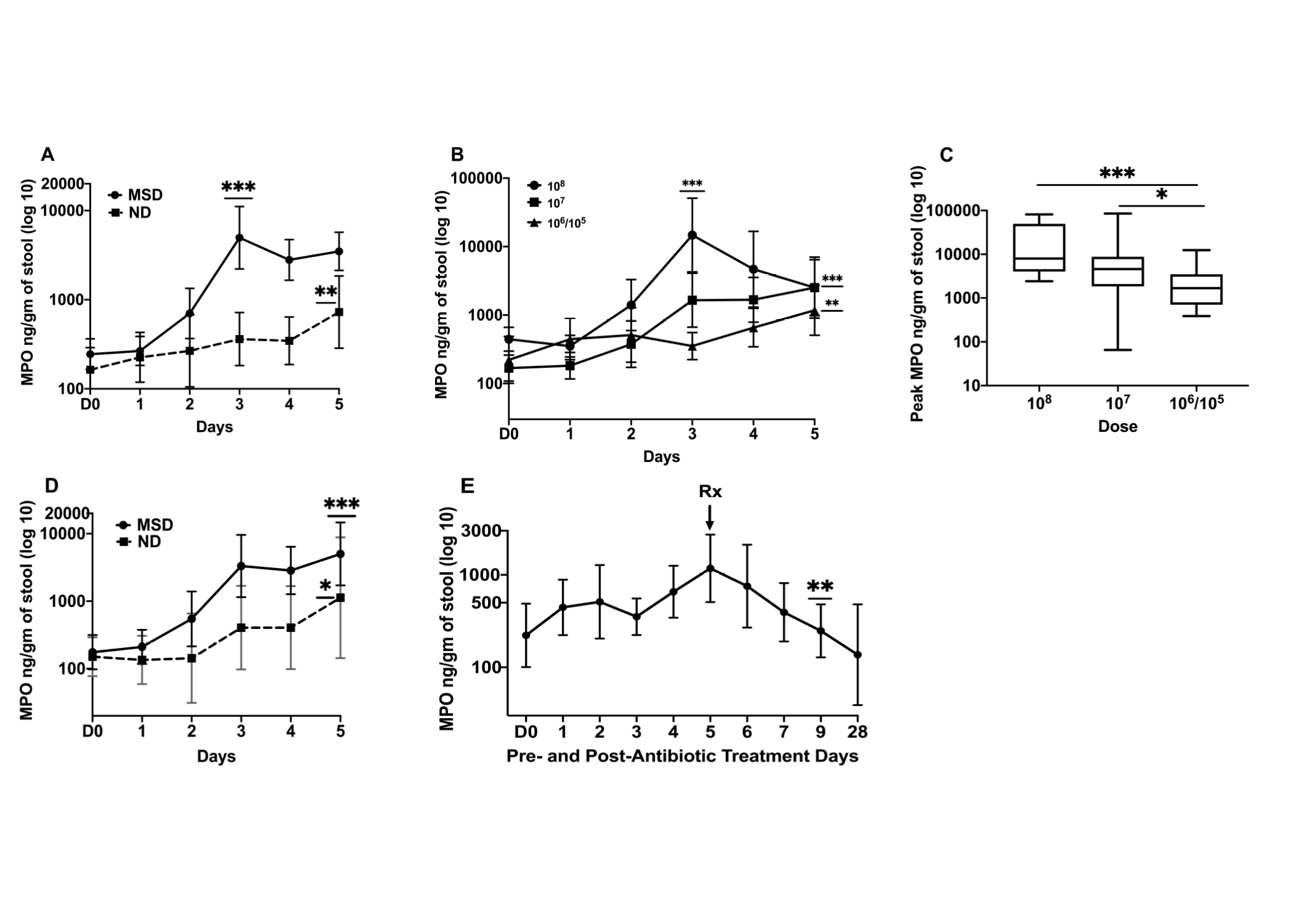

Supplement: Supplemental Material [file KGMI_A_1891852_SM3986.zip › Supplementary information/Supplement Fig 1.tif]

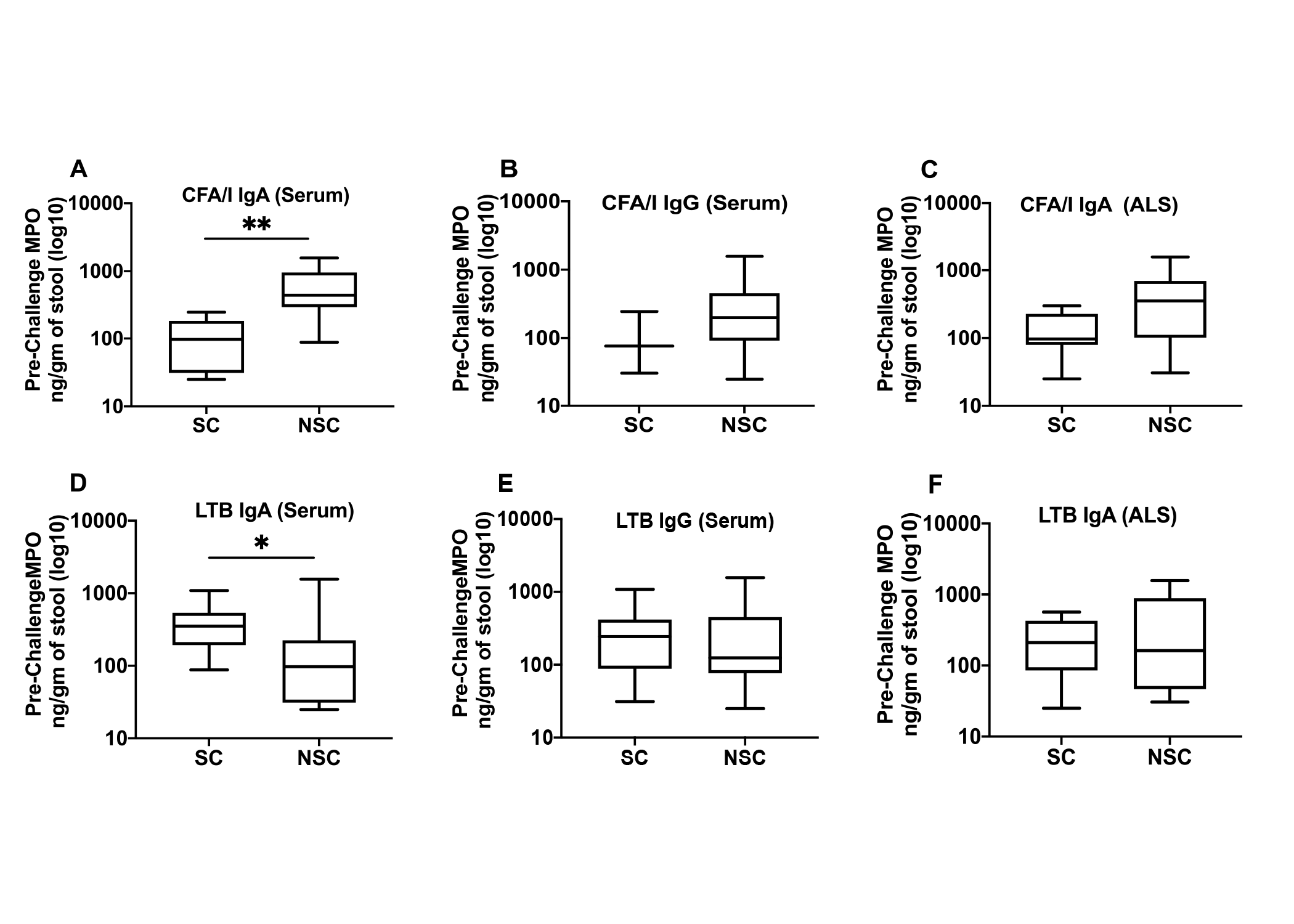

Supplement: Supplemental Material [file KGMI_A_1891852_SM3986.zip › Supplementary information/Supplement Fig 2.tif]
